# Supplementary material for: Interpreting the effects of DNA polymerase variants at the structural level
Source: Mol Oncol. 2026 May 27;20(8):2054–84. doi: 10.1002/1878-0261.70255 (PMC13398764; doi:10.1002/1878-0261.70255)
Supplement: Supplementary file 1 [file MOL2-20-2054-s002.pdf]

Figure S3. Top 25 VUS from the ranking protocol applied to the MAVISp data on variants with conflicting evidence from ClinVar for which MAVISp can identify structural alterations and VEPs predict damaging effects for POLD1. All the variants have been ranked using the most important features provided by MAVISp with associated weights as explained in Materials and Methods**.** The corresponding dotplots are reported in Figure 6 for an overview of the effects and the individual predictions.

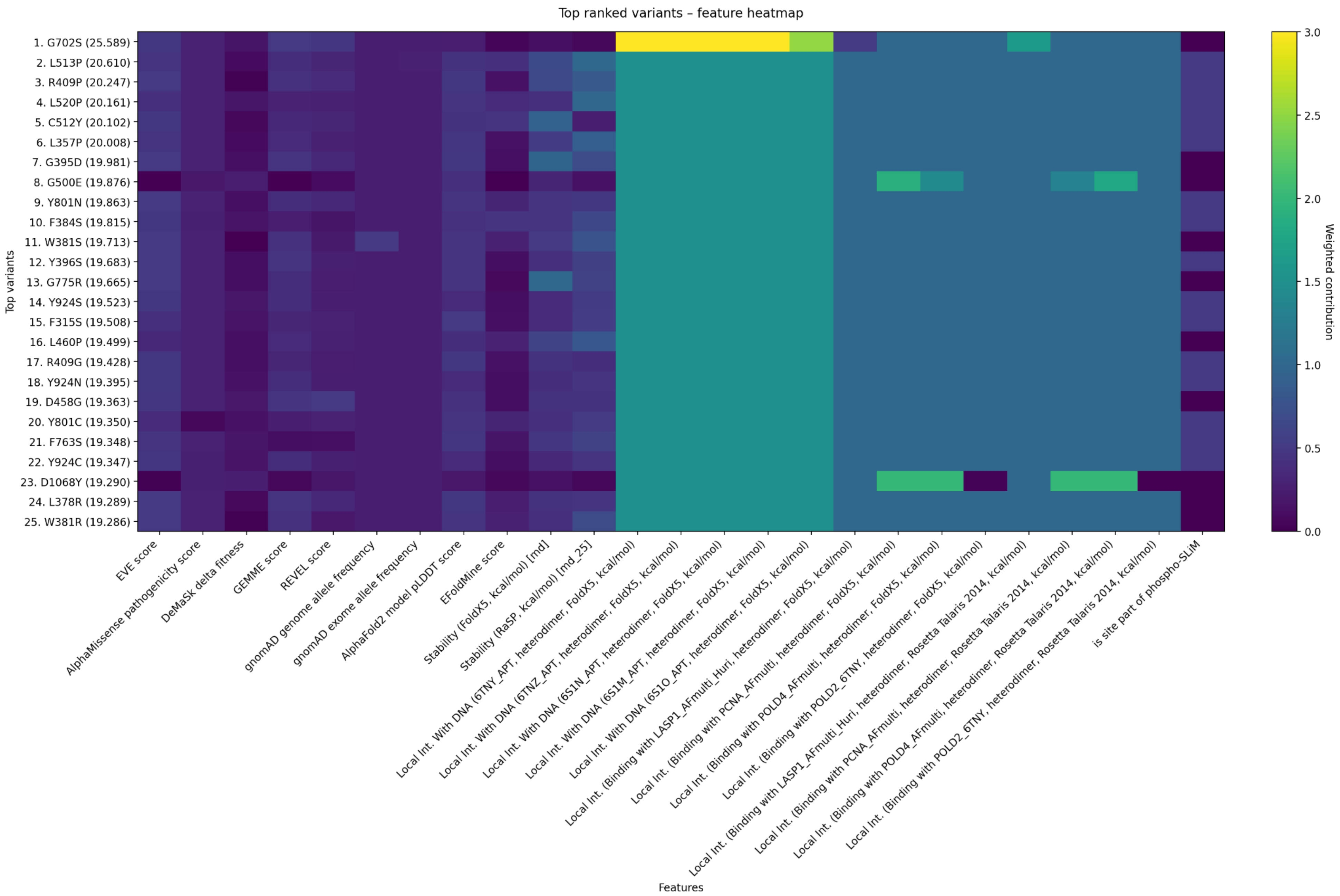
Top ranked variants – feature heatmap
Top variants
1. G702S (25.589)
2. L513P (20.610)
3. R409P (20.247)
4. L520P (20.161)
5. C512Y (20.102)
6. L357P (20.008)
7. G395D (19.981)
8. G500E (19.876)
9. Y801N (19.863)
10. F384S (19.815)
11. W381S (19.713)
12. Y396S (19.683)
13. G775R (19.665)
14. Y924S (19.523)
15. F315S (19.508)
16. L460P (19.499)
17. R409G (19.428)
18. Y924N (19.395)
19. D458G (19.363)
20. Y801C (19.350)
21. F763S (19.348)
22. Y924C (19.347)
23. D1068Y (19.290)
24. L378R (19.289)
25. W381R (19.286)
EVE score
AlphaMissense pathogenicity score
DeMaSk delta fitness
GEMME score
REVEL score
gnomAD genome allele frequency
gnomAD exome allele frequency
AlphaFold2 model pLDDT score
EFoldMine score
Stability (FoldX5, kcal/mol) [md]
Stability (RaSP, kcal/mol) [md_25]
Local Int. With DNA (6TNY_APT, heterodimer, FoldX5, kcal/mol)
Local Int. With DNA (6TNZ_APT, heterodimer, FoldX5, kcal/mol)
Local Int. With DNA (6S1N_APT, heterodimer, FoldX5, kcal/mol)
Local Int. With DNA (6S1M_APT, heterodimer, FoldX5, kcal/mol)
Local Int. With DNA (6S1O_APT, heterodimer, FoldX5, kcal/mol)
Local Int. (Binding with LASP1_AFmulti_Huri, heterodimer, FoldX5, kcal/mol)
Local Int. (Binding with PCNA_AFmulti, heterodimer, FoldX5, kcal/mol)
Local Int. (Binding with POLD4_AFmulti, heterodimer, FoldX5, kcal/mol)
Local Int. (Binding with POLD2_6TNY, heterodimer, FoldX5, kcal/mol)
Local Int. (Binding with LASP1_AFmulti_Huri, heterodimer, Rosetta Talaris 2014, kcal/mol)
Local Int. (Binding with PCNA_AFmulti, heterodimer, Rosetta Talaris 2014, kcal/mol)
Local Int. (Binding with POLD4_AFmulti, heterodimer, Rosetta Talaris 2014, kcal/mol)
Local Int. (Binding with POLD2_6TNY, heterodimer, Rosetta Talaris 2014, kcal/mol)
is site part of phospho-SLiM
Features
Weighted contribution
0.0
0.5
1.0
1.5
2.0
2.5
3.0
