# Supplementary material for: Interpreting the effects of DNA polymerase variants at the structural level
Source: Mol Oncol. 2026 May 27;20(8):2054–84. doi: 10.1002/1878-0261.70255 (PMC13398764; doi:10.1002/1878-0261.70255)
Supplement: Supplementary file 3 [file MOL2-20-2054-s008.pdf]

A

B

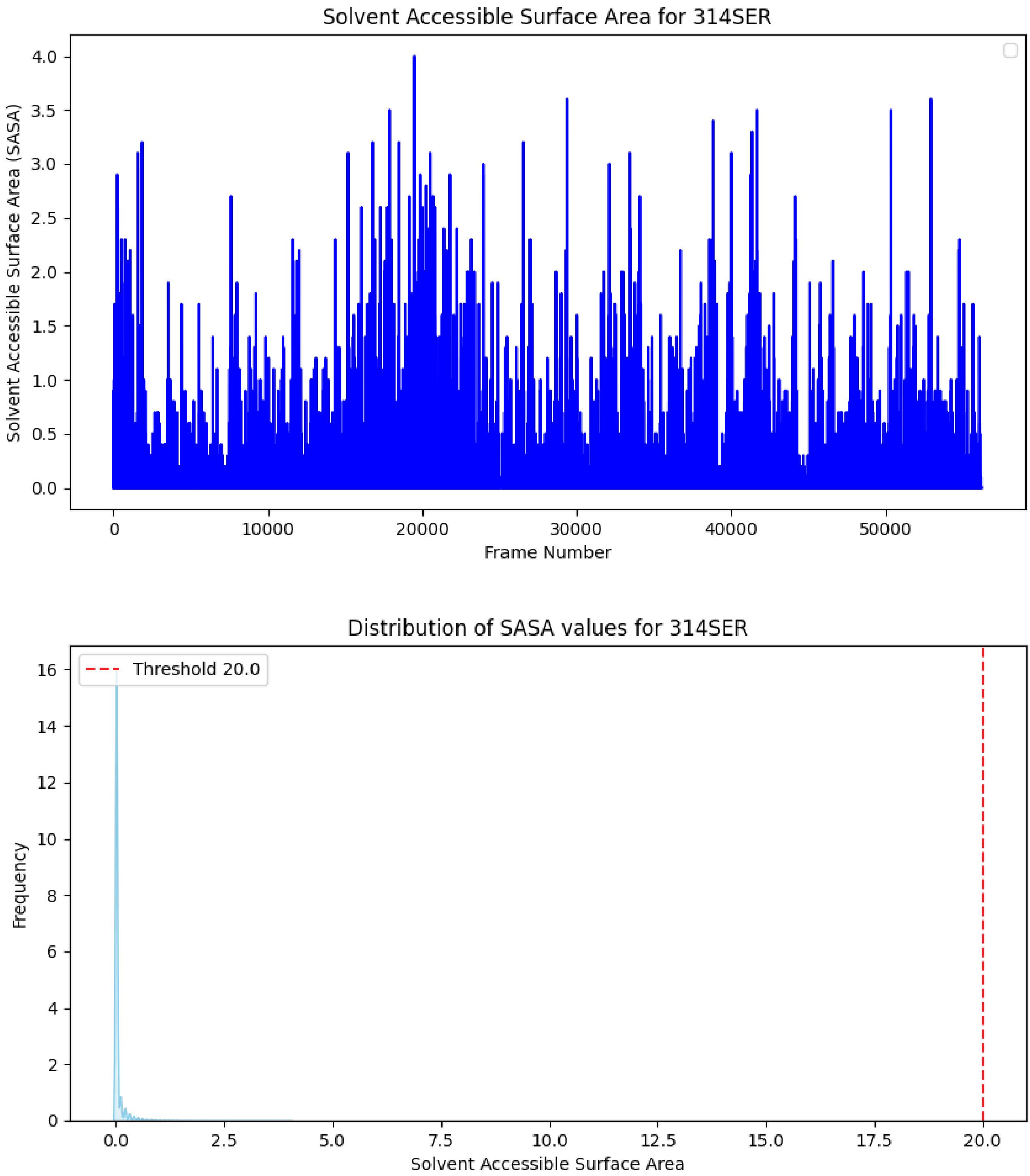


**Supplementary Figure S5. Solvent Accessibility (SASA) Analysis of S314 in POLD1.**
(A) Time series of the relative solvent accessibility of the side-chain atoms of S314 calculated from MD simulation frames sampled every 10 ps during the evolution of the POLD1 system.
(B) Density distributions of SASA values for the side-chain atoms of S314 computed across all the frames of the MD simulation.
