# Supplementary material for: Interpreting the effects of DNA polymerase variants at the structural level
Source: Mol Oncol. 2026 May 27;20(8):2054–84. doi: 10.1002/1878-0261.70255 (PMC13398764; doi:10.1002/1878-0261.70255)
Supplement: Supplementary file 5 [file MOL2-20-2054-s011.pdf]

Figure S2. Top 25 VUS from the ranking protocol applied to the MAVISp data on variants with conflicting evidence from ClinVar for which MAVISp can identify structural alterations and VEPs predict damaging effects for POLE. All the variants have been ranked using the most important features provided by MAVISp with associated weights as explained in Materials and Methods**.** The corresponding dotplots are reported in Figure 6 for an overview of the effects and the individual predictions.

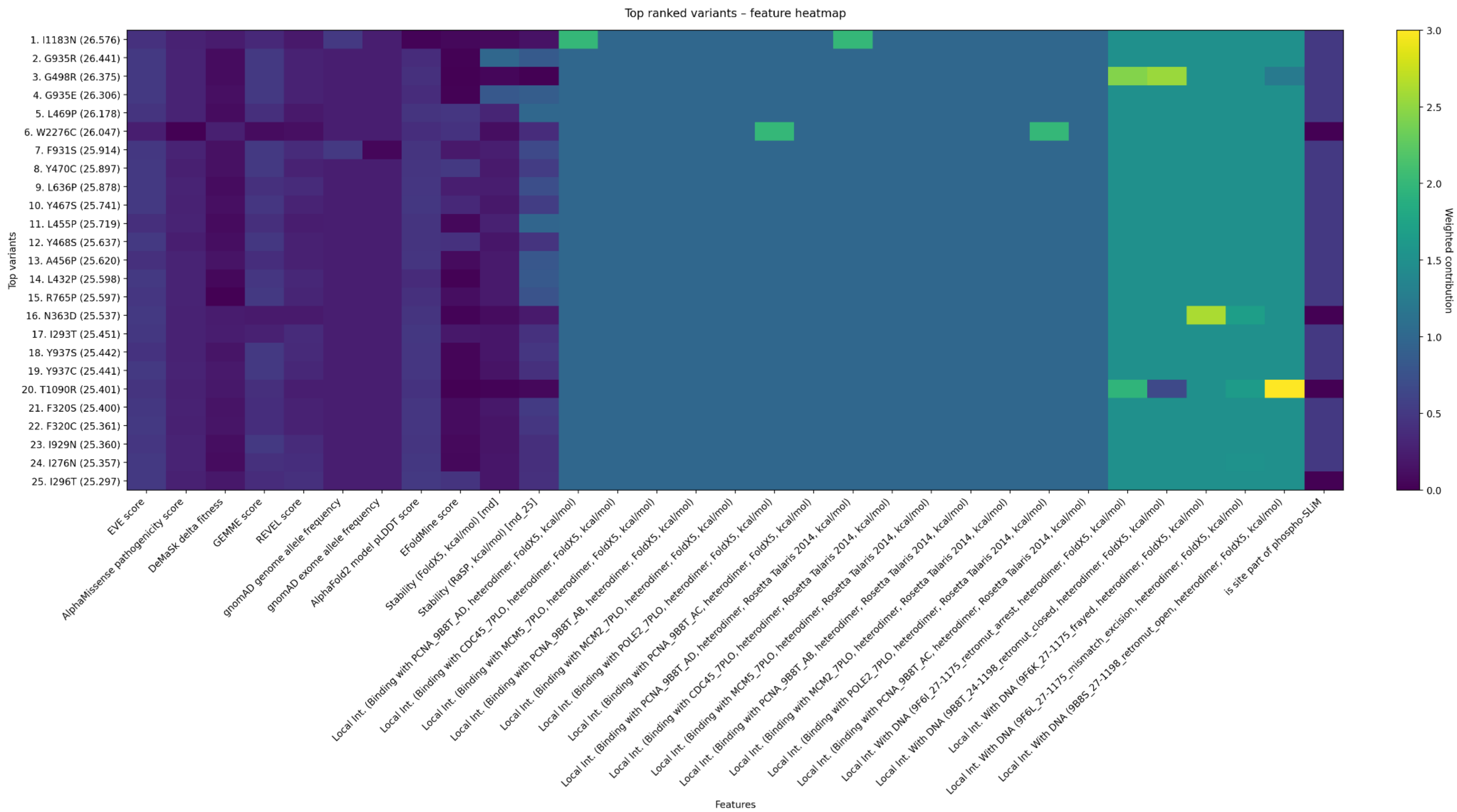

Top ranked variants – feature heatmap
Top variants
1. I1183N (26.576)
2. G935R (26.441)
3. G498R (26.375)
4. G935E (26.306)
5. L469P (26.178)
6. W2276C (26.047)
7. F931S (25.914)
8. Y470C (25.897)
9. L636P (25.878)
10. Y467S (25.741)
11. L455P (25.719)
12. Y468S (25.637)
13. A456P (25.620)
14. L432P (25.598)
15. R765P (25.597)
16. N363D (25.537)
17. I293T (25.451)
18. Y937S (25.442)
19. Y937C (25.441)
20. T1090R (25.401)
21. F320S (25.400)
22. F320C (25.361)
23. I929N (25.360)
24. I276N (25.357)
25. I296T (25.297)
Features
EVE score
AlphaMissense pathogenicity score
DeMaSk delta fitness
GEMME score
REVEL score
gnomAD genome allele frequency
gnomAD exome allele frequency
AlphaFold2 model pLDDT score
EFoldMine score
Stability (FoldX5, kcal/mol) [md]
Stability (RaSP, kcal/mol) [md_25]
Local Int. (Binding with PCNA_9B8T_AD, heterodimer, FoldX5, kcal/mol)
Local Int. (Binding with CDC45_7PLO, heterodimer, FoldX5, kcal/mol)
Local Int. (Binding with MCM5_7PLO, heterodimer, FoldX5, kcal/mol)
Local Int. (Binding with PCNA_9B8T_AB, heterodimer, FoldX5, kcal/mol)
Local Int. (Binding with MCM2_7PLO, heterodimer, FoldX5, kcal/mol)
Local Int. (Binding with POLE2_7PLO, heterodimer, FoldX5, kcal/mol)
Local Int. (Binding with PCNA_9B8T_AC, heterodimer, FoldX5, kcal/mol)
Local Int. (Binding with PCNA_9B8T_AD, heterodimer, Rosetta Talaris 2014, kcal/mol)
Local Int. (Binding with CDC45_7PLO, heterodimer, Rosetta Talaris 2014, kcal/mol)
Local Int. (Binding with MCM5_7PLO, heterodimer, Rosetta Talaris 2014, kcal/mol)
Local Int. (Binding with PCNA_9B8T_AB, heterodimer, Rosetta Talaris 2014, kcal/mol)
Local Int. (Binding with MCM2_7PLO, heterodimer, Rosetta Talaris 2014, kcal/mol)
Local Int. (Binding with POLE2_7PLO, heterodimer, Rosetta Talaris 2014, kcal/mol)
Local Int. (Binding with PCNA_9B8T_AC, heterodimer, Rosetta Talaris 2014, kcal/mol)
Local Int. With DNA (9F6I_27-1175_retromut_arrest, heterodimer, FoldX5, kcal/mol)
Local Int. With DNA (9B8T_24-1198_retromut_closed, heterodimer, FoldX5, kcal/mol)
Local Int. With DNA (9F6K_27-1175_frayed, heterodimer, FoldX5, kcal/mol)
Local Int. With DNA (9F6L_27-1175_mismatch_excision, heterodimer, FoldX5, kcal/mol)
Local Int. With DNA (9B8S_27-1198_retromut_open, heterodimer, FoldX5, kcal/mol)
is site part of phospho-SLiM
Weighted contribution
0.0
0.5
1.0
1.5
2.0
2.5
3.0
